# The Chaotic Behavior of the Spread of Infection during the COVID-19 Pandemic in Japan

**DOI:** 10.3390/ijerph191912804

**Published:** 2022-10-06

**Authors:** Nabin Sapkota, Atsuo Murata, Waldemar Karwowski, Mohammad Reza Davahli, Krzysztof Fiok, Awad M. Aljuaid, Tadeusz Marek, Tareq Ahram

**Affiliations:** 1 Department of Engineering Technology, Northwestern State University of Louisiana, Natchitoches, LA 71459, USA; 2 Department of Intelligent Mechanical Systems, Graduate School of Natural Science and Technology, Okayama University, Okayama 700-8530, Japan; 3 Department of Industrial Engineering and Management Systems, University of Central Florida, Orlando, FL 32816, USA; 4 Industrial Engineering Department, Taif University, Taif 26571, Saudi Arabia; 5 Department of Cognitive Neuroscience and Neuroergonomics, Institute of Applied Psychology, Jagiellonian University, 31-007 Kraków, Poland

**Keywords:** chaotic behavior, COVID-19 pandemic, the spread of infections, 0–1 test

## Abstract

In December 2019, China reported a new virus identified as SARS-CoV-2, causing COVID-19, which soon spread to other countries and led to a global pandemic. Although many countries imposed strict actions to control the spread of the virus, the COVID-19 pandemic resulted in unprecedented economic and social consequences in 2020 and early 2021. To understand the dynamics of the spread of the virus, we evaluated its chaotic behavior in Japan. A 0–1 test was applied to the time-series data of daily COVID-19 cases from January 26, 2020 to August 5, 2021 (3 days before the end of the Tokyo Olympic Games). Additionally, the influence of hosting the Olympic Games in Tokyo was assessed in data including the post-Olympic period until October 8, 2021. Even with these extended time period data, although the time-series data for the daily infections across Japan were not found to be chaotic, more than 76.6% and 55.3% of the prefectures in Japan showed chaotic behavior in the pre- and post-Olympic Games periods, respectively. Notably, Tokyo and Kanagawa, the two most populous cities in Japan, did not show chaotic behavior in their time-series data of daily COVID-19 confirmed cases. Overall, the prefectures with the largest population centers showed non-chaotic behavior, whereas the prefectures with smaller populations showed chaotic behavior. This phenomenon was observed in both of the analyzed time periods (pre- and post-Olympic Games); therefore, more attention should be paid to prefectures with smaller populations, in which controlling and preventing the current pandemic is more difficult.

## 1. Introduction 

Japan has experienced a widespread state of emergency due to COVID-19 three times since April 2020. The most populous city, Tokyo, and the Okinawa prefecture entered a 4th state of emergency (from 12 July until 22 August 2021). Three provinces surrounding Tokyo (Kanagawa, Chiba, and Saitama) were additionally declared to have a state of emergency from 2 to 31 July 2021. The central government asked the owners of restaurants that served liquor to close their business and the owners of restaurants that did not serve liquor to shorten their business hours and to close at 8 p.m. The state of emergency damaged the restaurant business and tourism [1,2,3,4]. Players and companies concerned from overseas were exceptionally permitted to enter Japan after a short quarantine period. To date, the 4th state of emergency appears not to have been effective in preventing the COVID-19 spread. 

Public health experts, including epidemiologists, strongly warned that holding the Olympic Games during the 4th state of emergency in Tokyo was not recommended because the predicted marked increases in SARS-CoV-2 infections, deaths, and hospitalized patients were expected to exceed the number of available sickbeds [5,6,7,8]. The experts were deeply concerned about safety and security in the Olympic Games during the state of emergency in Tokyo. Opinions opposing the Olympic Games were dominant among Japanese people [9], because many doubted whether a safe and secure Olympic Games would be possible under a state of emergency in Tokyo [10,11,12,13,14,15,16,17]. In contrast to the past three states of emergency, the number of infections has not decreased but has increased since the 4th declaration of a state of emergency in Tokyo. 

COVID-19 vaccination in Japan has been delayed compared to other developed countries, such as the US or UK, due to the regulatory requirements for a domestic clinical trial involving Japanese citizens and its review process [18]. As a result, the percentage of vaccination completion on 25 July 2021, was below approximately 25% [18,19].

The influence of the four states of emergency from April 2020 to the present across industries, particularly the restaurant business and tourism, has been tremendous. Consequently, many critiques have been made against the host city (Tokyo), the Japanese central government, the Olympic Organizing Committee (OOC), JOC, and IOC. The excess cost over the initially estimated expense of approximately USD 74 billion has been described by Zimbalist [20], Flyvbjerg, Budzier and Lunn [21], and Scandizzo [22]. 

The host city (Tokyo), the Japanese government, the OOC, JOC, and IOC have often been severely criticized for not providing evidence supporting their claim that a safe and secure Olympic Games were possible, and that SARS-CoV-2 infections could be kept to a minimum even under a state of emergency in Tokyo [10,11,12,13,14,15,16,17]. Although the Japanese people appeared to support the athletes and expected them to make achievements in the Olympic Games, they were skeptical about the safety and security of the Olympic Games because of the insufficient explanation for the reasons why the host city (Tokyo), the Japanese government, the OOC, JOC, and IOC believed that they could ensure the safety and security of the Olympic Games [23]. Tokyo’s governor asked residents to stay home and not to leave Tokyo to prevent increasing the cases of COVID-19 [24]; therefore, the JOC invited only the IOC directors to a welcome party and allowed only IOC members to leave Tokyo and visit Hiroshima and Nagasaki [25,26].

As described above, the Olympic Games during the state of emergency were expected to not be safe and secure under the 4th state of emergency. This study attempted to assess the severity of widespread COVID-19 on the basis of deterministic chaos by using the K-median values of the 0–1 test for a time-series of infections before, during, and after the states of emergency (1st, 2nd, 3rd, and 4th). On the basis of this analysis, the effects of the Olympic Games on safety and security under widespread COVID-19 conditions were assessed.

### 1.1. Objectives

To investigate the chaotic behavior of the COVID-19 pandemic, we analyzed data from a total of 47 prefectures of Japan. Specifically, we aimed to confirm that the COVID-19 epidemic displayed chaotic behavior, according to the analysis of time-series data of the reported daily confirmed cases. First, we investigated the spread of daily confirmed cases in various Japanese prefectures, then examined their behavior in the country as a whole. The present article is structured as follows: the background section discusses the objectives and conclusions of published articles concerning the chaotic behavior of pandemics; the methods section explains the binary 0–1 test for chaos using time-series data; the results section represents the outcomes of the 0–1 test; and the discussion section explains the results.

### 1.2. Background

On December 8, 2019, China reported multiple new virus cases identified as coronavirus disease 2019 (COVID-19) [27,28]. Since then, the SARS-CoV-2 virus has spread from China to many countries and caused a global pandemic [27,28]. The first confirmed case of SARS-CoV-2 infection in Japan was reported on 16 January 2020, in a Chinese national who had returned from China [29]. After the identification of this confirmed COVID-19 case, schools were closed on 27 February 2020 [29]. By the end of February 2020, several confirmed COVID-19 cases had been identified nationwide [30]. In mid-March, the number of cases had increased significantly, an effect potentially associated with returnees and travelers from North America and Europe [30]. On 16 April 2020, the Japanese government declared a state of emergency for the entire country [29,31].

In theory, Japan was very vulnerable to the pandemic for several reasons including (1) the high travel volumes between Japan and China; (2) the proximity of the two countries; (3) the high population density in large cities; (4) the high number of older people; and (5) the high volumes of commuters [32]. However, Japan was able to control the initial spread of the COVID-19 pandemic and reduce the number of confirmed cases and deaths [33]. Several studies have tried to explain the low mortality rate in Japan during the pandemic. One study has indicated that Japanese people might have relative immunity due to exposure to a milder strain of the COVID-19 or due to the mandatory Bacille Calmette–Guerin tuberculosis vaccine [34]. Another study has related the low mortality rate to Japanese cultural traits such as bowing and avoiding handshakes, removing shoes, social distancing, and the use of face masks [30,34]. One study has argued that the combination of multiple elements including food habits, basic sanitation practices, Japan’s basic health policy, and individuals’ health consciousness might explain the low mortality rate [32]. Nonetheless, analyzing the governmental responses can enable a better understanding of the behavior of the pandemic in Japan.

The Japanese government focused on identifying common sources of infection by developing a cluster-based response [30] through the perspective and retrospective tracing of infection. The main response strategies were (1) early identification and action toward infected clusters, (2) early detection of critical patients and early allocation of intensive care and medical services, (3) securing intensive care and medical service systems for ill patients, and (4) the modification of individual behaviors and prevention of environmental transmission in crowded places, closed spaces, and close-contact settings. This response has been reported to have been effective in the early stages of the pandemic when infected clusters could be prevented from spreading into new clusters [32]. With the increasing number in confirmed cases in March and April 2020, cluster tracing became difficult, because many cases were associated with pubs, sports clubs, and other night entertainment. Therefore, the Japanese government developed a more comprehensive response containing multiple stages including containment (reducing the number of returnees from key affected areas), medical service reinforcement (strengthening medical capacity and increasing testing capacity), mitigation (establishing the Basic Countermeasure Policy based on recommendations from the expert committee), emergency (providing a stronger legal basis for countermeasures), and recovery (focusing on the recovery of the economy) [32].

Overall, the behavior of the COVID-19 pandemic in Japan can be divided into five waves (peaks) including the first wave from 26 January to 31 May 2020; the second wave from July to September 2020; the third wave from October 2020, with a peak in January 2021 [29,35]; the fourth wave in May to June 2021; and the fifth wave from July to September 2021. Whereas the first wave had a peak of 701 new confirmed cases per day, the second wave peaked at 1762 new cases per day [36]; however, the third wave exceeded the previous waves, with 8045 new cases per day. The fourth wave had a peak of 7239 cases per day, whereas the fifth wave had a peak of 25,851 cases per day on 20 August 2021, 12 days after the conclusion of the Tokyo Olympics. The main reasons for this rise included a return to normal life, the opening of certain businesses, and relaxed immigration restrictions. The main problem in the third wave was that the bed usage for patients exceeded 50% nationwide [36]. The Japanese Government prepared its medical system with approximately 19,000 rooms and 18,000 beds being available nationwide for patients with critical COVID-19 [36].

One way to better understand the behavior of the pandemic in Japan is to compare Japan with other countries. For example, Japan is lifting or re-imposing restrictions based on epidemiological thresholds such as those in Germany and South Korea [37]. In general, centralized countries, such as China, with homogeneous governance structures, can easily implement more stringent response policy measures than can decentralized countries, such as Japan, which prefer providing recommendations [38]. One study comparing Japan with the US has indicated that Japan was more successful at controlling the pandemic [39]. For example, on 1 August 2020, the number of new daily confirmed cases was 6.3 in Japan and 194 in the US; on 20 July 2020, the number of deaths per 100,000 population was 0.78 in Japan and 49.5 in the US. The study focused on four aspects of pandemic governance: the culture of mask-wearing, the social value of following governmental advice, the role of national leadership, and the clarity of the provided information to explain this comparison. Another study has compared Japan with Germany and indicated that Japan was more successful in reducing the speed of spread than Germany during the states of emergency [29]. The study has described that Germany experienced a much larger number of confirmed cases than Japan, possibly because of geographic differences (e.g., island vs. continental Europe).

Although Japan was relatively successful in controlling the pandemic, several problems existed regarding its response to the pandemic. First, Japan was not successful in expanding laboratory testing. During the early stages of the outbreak, the cumulative number of COVID-19 tests in Japan was less than that in other countries [30]. This lack of testing might have led to an increase in the number of undiagnosed COVID-19 cases. Second, the healthcare system in Japan relies on paperwork; thus, resulting in inefficiency and inaccuracies [40]. Third, the government did not encourage people to change their behavior during the states of emergency [40]. Fourth, a lack of accountability and transparency in government existed regarding the postponing of the Tokyo 2020 Olympics and the COVID-19 decision-making processes.

Another aspect of the pandemic in Japan is the rate of COVID-19 vaccine acceptance. One cross-sectional study based on an internet survey has indicated that approximately 62% of participants indicated that they would get the COVID-19 vaccine [41]. The study has reported that the rate of vaccine acceptance is lower among people with a low income, women, and adults 20–49 years of age.

### 1.3. Literature Review

Similarities exist between the dynamics of pandemic spread and other well-known nonlinear systems, such as turbulent flow [42]. In nonlinear systems, a small seed grows exponentially and finally saturates. Because of this similarity, some features of nonlinear behavior, such as chaos, can be used to better understand pandemics [43]. Chaotic behavior shows the sensitivity of a system to the initial conditions and many studies have analyzed chaotic behavior in infectious disease spread. For example, one study developed a compartmental model (e.g., susceptible infected with multi-drug resistance) to determine the COVID-19 pandemic spreading patterns [44]. The study used various numerical tools of Lyapunov exponents (LEs), bifurcation diagrams, and Lyapunov spectra to investigate the complex dynamics of the model. An LE was applied to determine the chaotic behavior of a model by determining the divergence rate of trajectories in the phase space [45]. Whereas an LE above zero (positive) may represent chaotic behavior, an LE less than zero (negative) does not generally indicate stability.

Beyond the COVID-19 pandemic, chaotic behavior has been investigated in other pandemics such as the Rift Valley fever epidemic [46], childhood diseases [47], measles epidemics [48], and epizootic outbreaks [49]. Several articles have studied the chaotic behavior of pandemics under specific conditions, such as the presence of seasonality [50,51], the presence of noise [50], the discrete-time and continuous-time epidemic model [52], and different locations [48]. For example, Grenfell et al. [48] have developed a time-series-susceptible-infected-removed model to investigate the chaotic behavior of measles epidemics in several cities. The study concluded that small cities are more stable than large cities.

In all the described articles, an LE has been used to determine the chaotic behavior, and an LE > 0 indicates chaos in a pandemic spread [43]. However, a positive LE may not be a strong indicator of chaos in a system [53]. Recently, several studies have used the binary 0–1 test to confirm the chaotic behavior of pandemics. For example, one study has developed a susceptible-exposed-infectious-removed model for the COVID-19 pandemic and used the 0–1 test to confirm chaos in the presence of introduced seasonality and stochastic spreading [54]. Another study has developed susceptible/resistant-antimicrobial resistance models and analyzed complex dynamics such as chaotic behavior, flip bifurcations, the existence of homoclinic connections, and the coexistence of multiple attractors. The study used a 0–1 test to confirm chaos in the models [55].

## 2. Methods

This section outlines the methods for the determination of deterministic chaos using the 0–1 test and discusses relevant SARS-CoV-2 infection time-series data.

### 2.1. Determination of Deterministic Chaos with the 0–1 Test

The applied 0–1 test procedure was based on the notation outlined by Gottwald et al. [56] and has been fully explained in the Appendix A of our previous publication, Sapkota et al. [43].

### 2.2. COVID-19 Data Source and Processing

The data used in the present study were collected from the Japan Ministry of Health, Labour, and Welfare (MHLW) COVID-19 Data [57]. These data are a part of the ministry’s effort to inform the public regarding SARS-CoV-2 infections in the country in real time. At the time of the data retrieval, these data were among the many featured on the webpage titled ‘Visualizing the data: information on SARS-CoV-2 infections’ at (https://covid19.mhlw.go.jp/en/ accessed on 10 August 2021). The name of the data file used for this study was newly_confirmed_cases_daily.csv. This is an active file and is updated regularly as data becomes available.

The data processing was performed with pandas (ver. 1.3.0 Snyk (Berkshire, England)), an open-source data analysis and manipulation library in the Python programming language. These data contain newly confirmed SARS-CoV-2 infection cases for the entire country and Japanese cities/prefectures between 26 January 2020, and 5 August 2021 (3 days before the end of the Tokyo Olympic Games). Table 1 shows a sample of the top five and bottom five records from the data.

The data in Table 1 contained 26,1784 records that were processed to obtain time-series data for each prefecture between 26 January 2020 and 5 August 2021, including one for the entire country. After data processing, 48 time-series were created (one each for 47 prefectures and one for the entire country) for the daily newly confirmed cases of SARS-CoV-2 infection.

## 3. Results

The results of the 0–1 test for chaotic behavior of the spread of SARS-CoV-2 infection based on the daily count of confirmed cases in Japan and its prefectures are discussed below.

### 3.1. Spread of SARS-CoV-2 Infection in Japan

For Japan, the time-series data for each prefecture from the day of the first confirmed case (SARS-CoV-2 infection) were used. The K-median values from the 0–1 test were used to signify whether the time-series infection data exhibited deterministic chaos. The time-series data with K-median values at or greater than 0.9 were classified as chaotic, and those with K-median values less than 0.9 were classified as non-chaotic.

**Figure 1:**
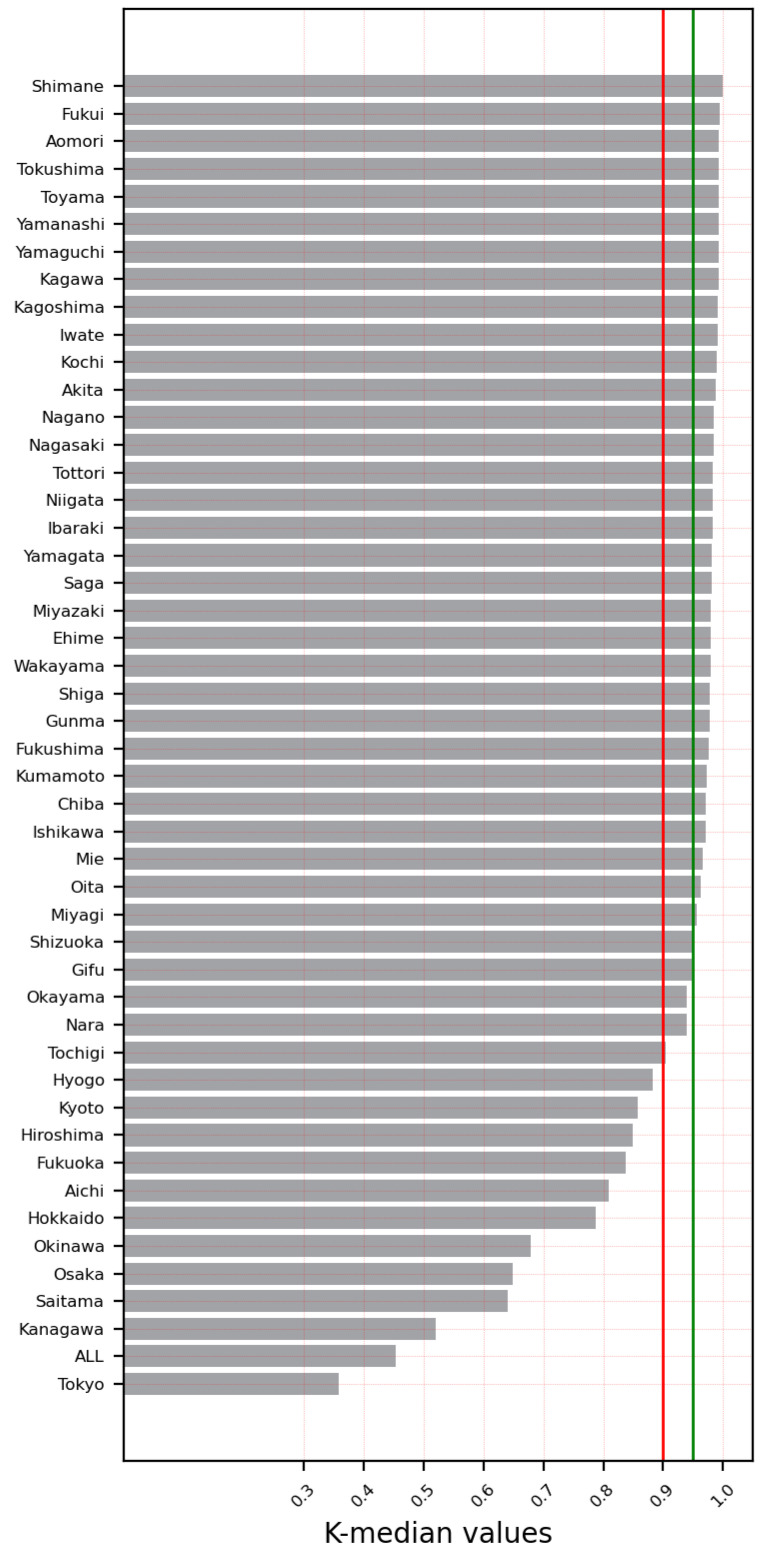
K-median values for all prefectures including the entire country (data until 5 August 2021).

**Figure 2:**
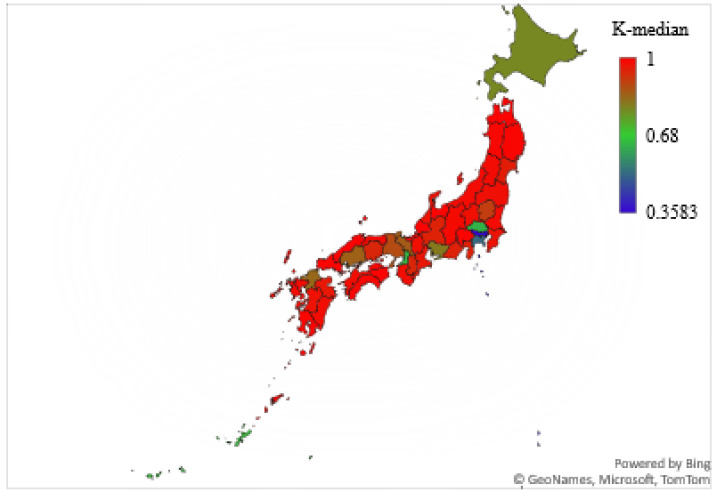
K-median values from 0–1 test for cities/prefectures of Japan.

The results showed that the spread of SARS-CoV-2 infection in 36 of 47 prefectures was chaotic (76.6%). The cities/prefectures that did not show chaotic behavior in the examined time-series data for daily infections were Aichi, Fukuoka, Hiroshima, Hokkaido, Hyogo, Kanagawa, Kyoto, Okinawa, Osaka, Saitama, and Tokyo. Table 2 and Figure 1 show the K-median values from the 0–1 test for the confirmed daily COVID-19 cases by Japanese cities/prefectures. In addition, Figure 2 illustrates a geographical map of Japan with prefectures, with the dark red color signifying the chaotic behavior of the time-series data representing the daily spread of infections.

**Figure 3:**
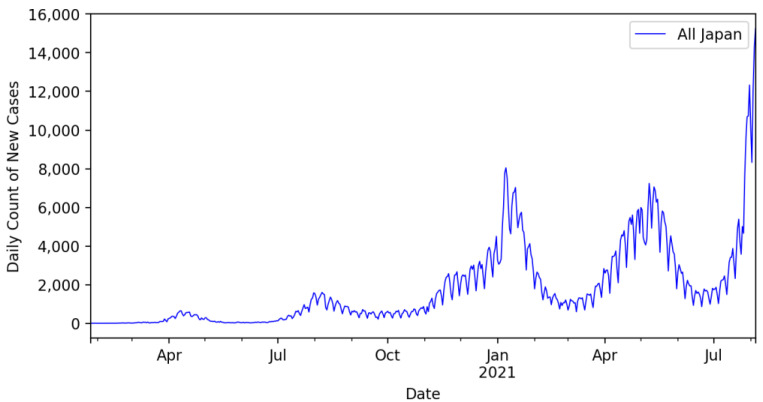
Daily confirmed cased of COVID-19 in Japan from January 2020 to July 2021.

The study indicated that most prefectures in Japan showed chaotic behavior in the daily new infection cases; however, analysis of the overall new daily infection cases for the country indicated that the K-median values were approximately 0.4536. Because this value is not close to 1, as the test required to confirm the chaotic behavior in the time-series data of the daily count of newly infected cases, Japan cannot be ascertained to be a country with a chaotic spread of COVID-19. The overall daily infection counts for the entire country for the duration considered in this study are shown in Figure 3. In Figure 3, the daily count of the confirmed COVID-19 cases shows multiple peaks (mid-April 2020, August 2020, January 2021, and mid-June 2020). The last two peaks were approximately similar in value and were much higher than the first two peak values. The daily count approached another all-time peak value in August 2021 (12,329 confirmed cases in the country on 31 July 2021 and 15,249 confirmed cases on 5 August 2021).

**Figure 4:**
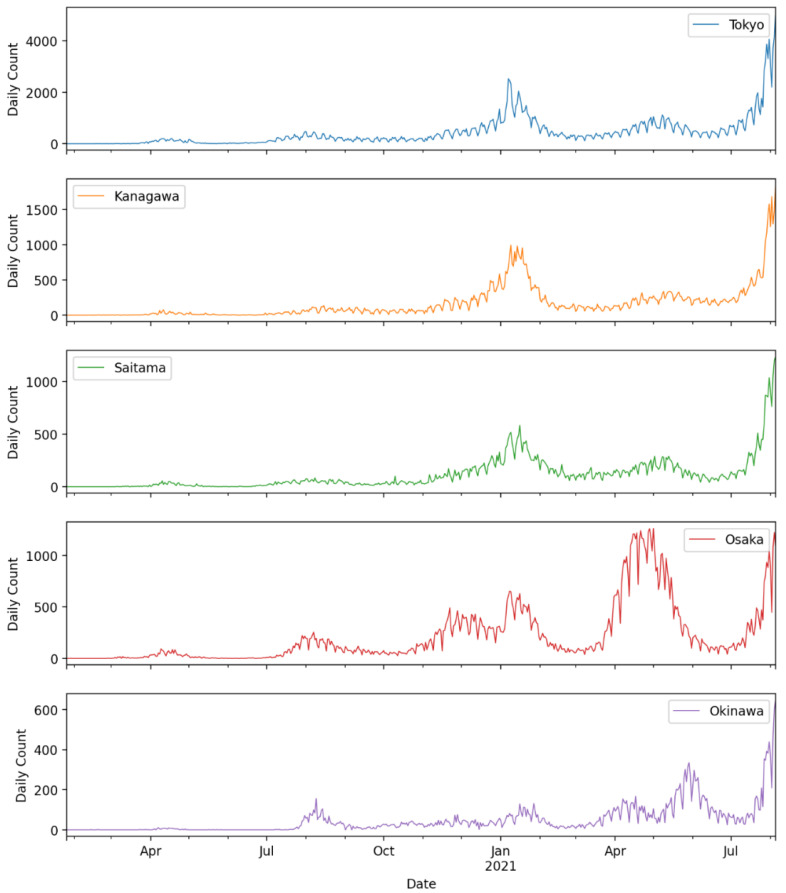
Bottom five prefectures with the lowest K-median values.

**Figure 5:**
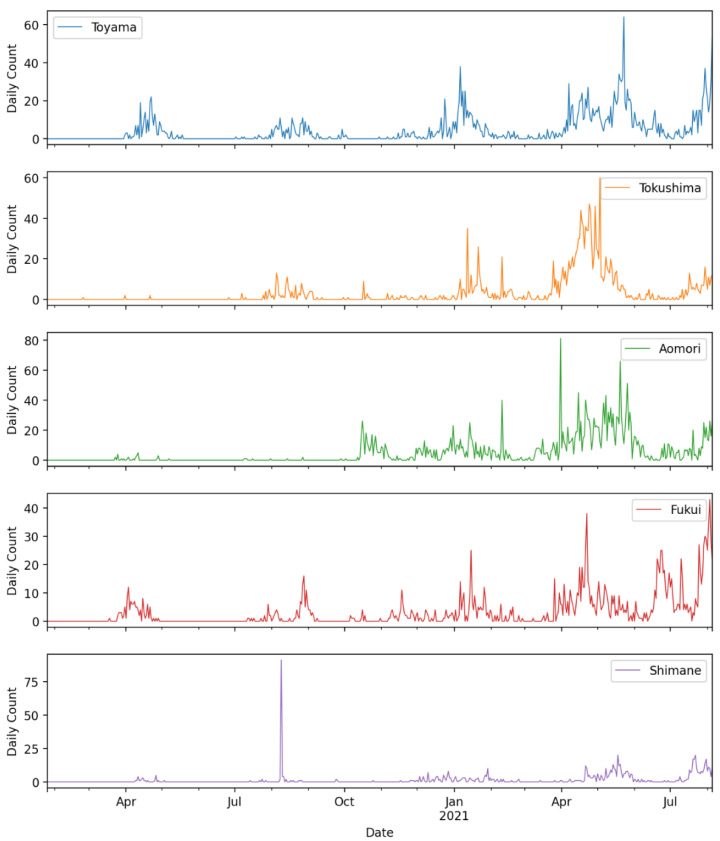
Top five prefectures with the highest K-median values.

Figure 4 shows the daily confirmed COVID-19 cases in prefectures with the lowest K-median values for the duration of the data used in this study. Similarly, Figure 5 shows the daily confirmed cases of the COVID-19 cases in prefectures with the highest K-median values for the duration of the data used in this study.

### 3.2. Spread of SARS-CoV-2 Infection after the Tokyo Olympics

To analyze the after-effects of the Tokyo Olympic Games, we added data from August 6 to 8 October 2021 to the previous data. The data were processed in a similar manner to those of the previous case.

**Figure 6:**
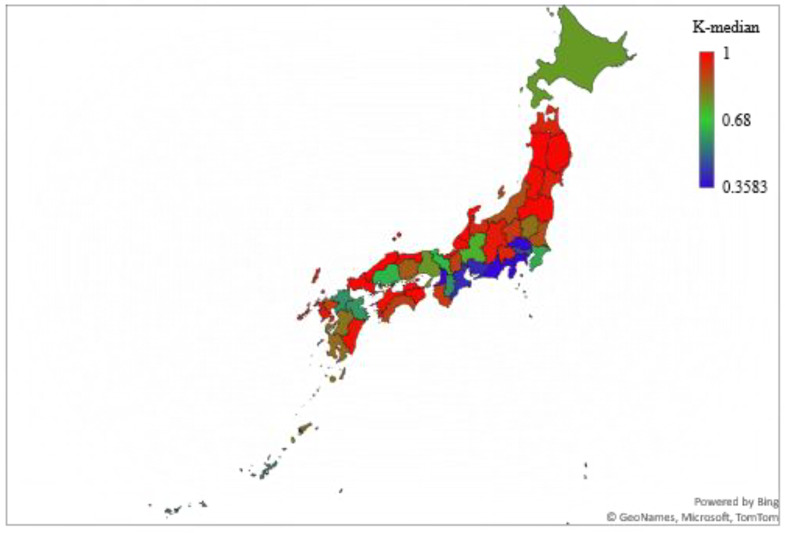
K-median values from 0–1 test for cities/prefectures of Japan for data until 8 October 2021.

**Figure 7:**
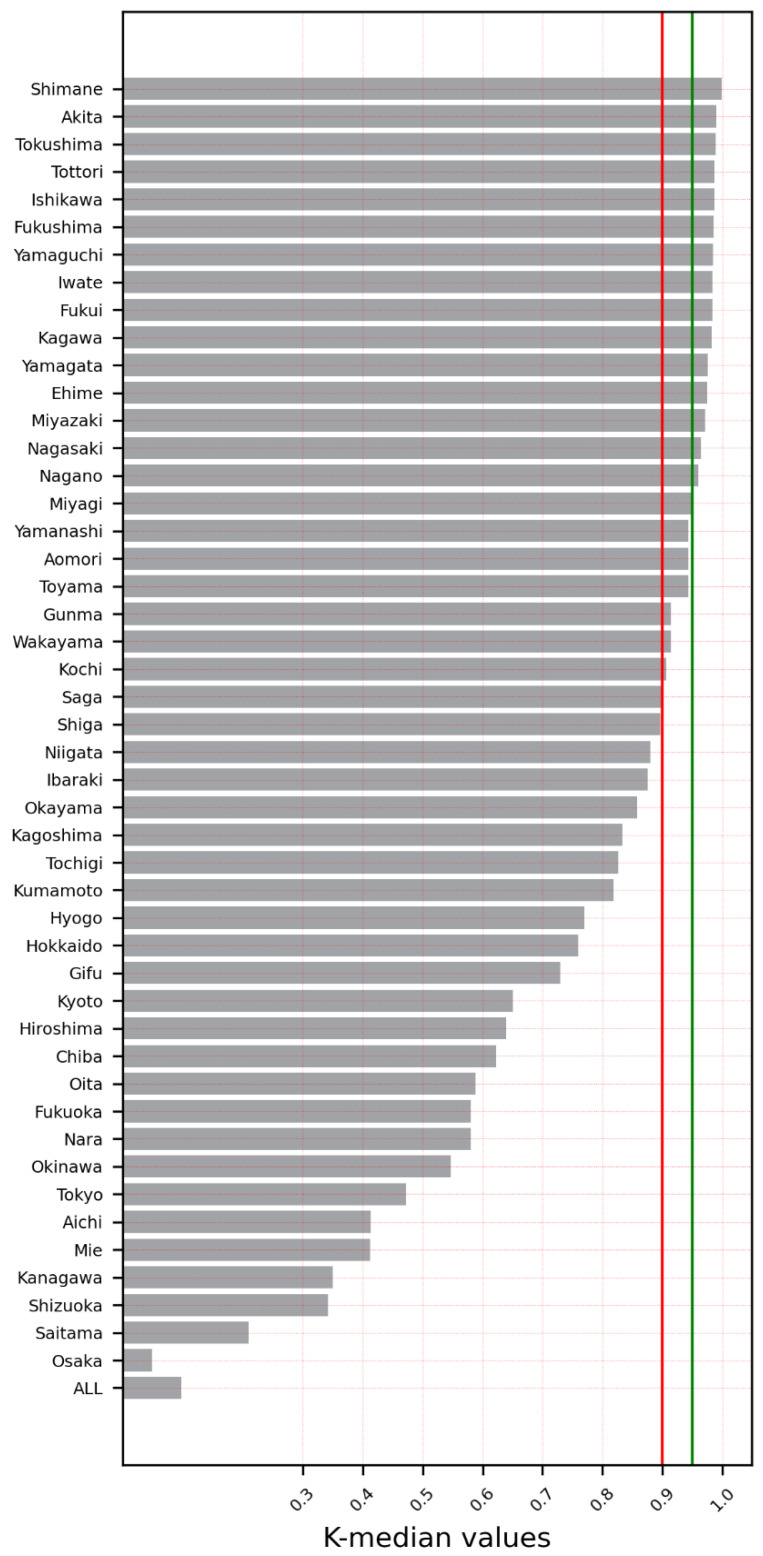
K-median values for all prefectures including the entire country for data until 8 October 2021.

Figure 6 illustrates a geographical map of Japan with its prefectures, with the dark red color signifying the chaotic behavior of the time-series data representing the daily spread of infections until 8 October 2021. In addition, Figure 7 shows the K-median values from the 0–1 test for the confirmed daily COVID-19 cases in Japanese cities/prefectures for the same duration. One notable shift was observed for Chiba prefecture, which displayed chaotic behavior in the data pre-Olympic Games but displayed non-chaotic behavior in the full data (until 8 October 2021), with a K-median value of 0.62. Chiba is one of the three prefectures that surrounds Tokyo, the host city of the Olympic Games. Furthermore, the top five K-median values were found to be for Shimane, Akita, Tokushima, Tottori, and Ishikawa, whereas the bottom five K-median values were observed for Mie, Kanagawa, Shizuoka, Saitama, and Osaka.

The results showed that the spread of SARS-CoV-2 infection in 27 of 47 prefectures was chaotic (55.3%), and a decrease was observed in 19.1% of the prefectures. All 11 cities/prefectures that did not show chaotic behavior in the time-series data in the first analysis (until 5 August 2021) also showed the same behavior. Several additional cities/prefectures were included in the post-Olympics analysis. These cities were Shiga, Niigata, Ibaraki, Okayama, Kagoshima, Tochigi, Kumamoto, Gifu, Kyoto, Chiba, Oita, Nara, Mie, and Shizuoka.

**Figure 8:**
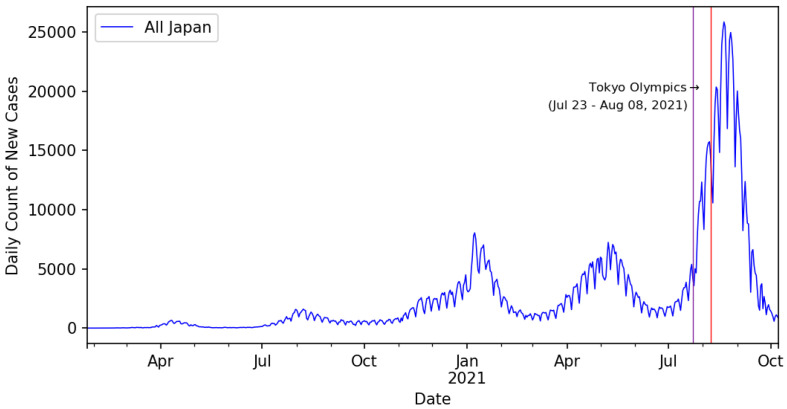
Daily count of new cases in all Japan for data until 8 October 2021.

Figure 8 shows the daily new cases count for the entire period for all Japan. Before the start of the Olympic Games (23 July 2021), the country was already heading toward the fifth wave. As described earlier, the fifth wave peaked on 20 August 2021, with 25,851 new daily confirmed cases.

**Figure 9:**
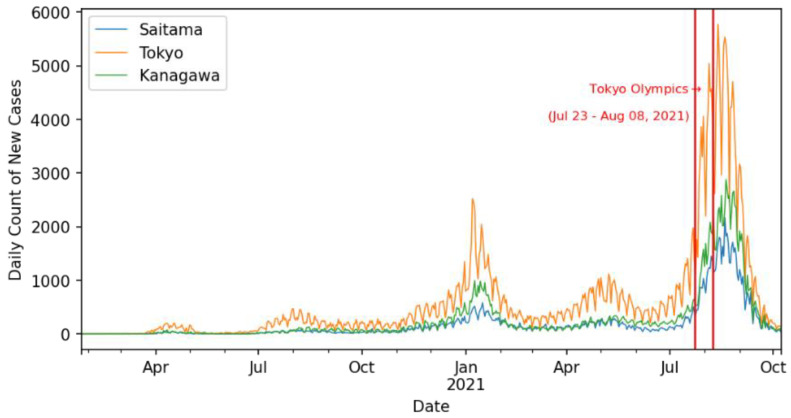
Daily count of new cases in Tokyo and surrounding prefectures for data until 8 October 2021.

This finding might be argued to be a result of additional activities and the influx of people (e.g., athletes, foreign officials, and organizing staff) in preparation for the Olympic Games before their start on 23 July 2021. Around the peak time of the fifth wave (20 August 2021), 40% of the daily count of new confirmed cases was contributed by the Tokyo Metropolitan area (Tokyo, Kanagawa, Saitama, and Chiba) and the rest of the 60% of the daily count was contributed by the rest of the prefectures in Japan. Moreover, the prefectures of Tokyo, Kanagawa, and Saitama never displayed chaotic behavior in the time-series data. Figure 9 shows daily count of new cases for Tokyo, Kanagawa, and Saitama for the period of this study as well as the duration of the Olympic games (vertical red lines). 

## 4. Discussion

In our previous study, we concluded that, although the data for the daily confirmed cases for the entire US were not found to be chaotic, more than 35% of the states showed chaotic behavior [43]. On the global scale, the results of our previous study indicated that the dynamics of the pandemic was chaotic in more than 55% of countries [43]. The behavior of the virus was not chaotic on a country scale in Italy, Spain, or even the United Kingdom [43].

The results obtained in this study are consistent with the results of our previous publication. In the present study, only the confirmed daily COVID-19 cases in Japan and its prefectures were subjected to a nonlinear dynamics analysis. The available data for the number of hospitalizations, the daily number of deaths, and number of recoveries were not considered here. Although the time-series data for the daily infections in the entirety of Japan were not found to be chaotic, the results of the prefecture-level analysis indicated that the confirmed SARS-CoV-2 infections for more than 76.6% of prefectures showed chaotic behavior. Additionally, the influence of hosting the Olympic Games in Tokyo was assessed by an investigation of data including the post-Olympic Games period until 8 October 2021. Even with the extended time period, although the time-series data for the daily infections throughout Japan were not found to be chaotic, 55.3% of its prefectures, nonetheless, showed chaotic behavior in the post-Olympic Games period. Notably, the Tokyo and Kanagawa prefectures include the two most populous cities in Japan, namely, Tokyo and Yokohama. These two cities did not show chaotic behavior in their time-series data of daily COVID-19 confirmed cases. Moreover, these two prefectures/cities are next to each other. In addition, Tokyo is sandwiched between the Kanagawa and Saitama prefectures, and both prefectures showed non-chaotic behavior in their respective time-series data.

We additionally observed that the prefectures with the largest population centers showed non-chaotic behavior, whereas the prefectures with smaller populations showed chaotic behavior.

Multiple limitations of the methods and results should be acknowledged. First, this article did not consider several factors that may play important roles in determining the dynamics of the spread of infection, such as socioeconomic and demographic factors, including social customs and traditions, education, income, employment, total population, the proportion of individuals under the poverty line, urban vs. rural populations, or population density. Second, data concerning government regulations, sociopolitical factors, and public health policies should also have been included to clarify the nonlinear behavior of the pandemic. Only by considering these data could effective countermeasures be developed. Third, to complement the current results, the largest Lyapunov exponent of each time-series for daily confirmed cases should also be computed, which may provide insight into the time-series data to understand immediate future behavior within the prediction horizons, as identified through the largest Lyapunov exponents.

## 5. Conclusions

In this article, time-series data indicating the spread of SARS-CoV-2 infection according to the daily confirmed cases from 26 January 2020 to 5 August 2021 (3 days before the end of the Tokyo Olympic Games) were analyzed for the presence of chaos. In more than 76.6% of the prefectures in Japan, the spread of SARS-CoV-2 infection was chaotic. Chaotic behavior makes the dynamics of a pandemic more difficult to determine and predict in the long term [30].

Our results indicate the importance of considering chaotic behavior in public health policies. As already described, other important data concerning government regulations and sociopolitical factors should be included to increase the understanding of the nonlinear behavior of SARS-CoV-2 infection in Japan and to develop efficient countermeasures.

## Figures and Tables

**Table 1:** Sample of raw data.

	Date	Prefecture	Newly Confirmed Cases
First five records	26 January 2020	All	1
	26 January 2020	Hokkaido	0
	26 January 2020	Aomori	0
	26 January 2020	Iwate	0
	26 January 2020	Miyagi	0
Last five records	5 August 2021	Kumamoto	127
	5 August 2021	Oita	31
	5 August 2021	Miyazaki	36
	5 August 2021	Kagoshima	51
	5 August 2021	Okinawa	648

**Table 2:** K-median values for all prefectures of Japan for daily confirmed COVID-19 cases.

Prefecture	K-Median
Tokyo	0.3583
Kanagawa	0.5200
Saitama	0.6402
Osaka	0.6484
Okinawa	0.6793
Hokkaido	0.7869
Aichi	0.8094
Fukuoka	0.8375
Hiroshima	0.8498
Kyoto	0.8577
Hyogo	0.8828
Tochigi	0.9040
Nara	0.9397
Okayama	0.9401
Gifu	0.9472
Shizuoka	0.9486
Miyagi	0.9556
Oita	0.9632
Mie	0.9661
Ishikawa	0.9705
Chiba	0.9709
Kumamoto	0.9728
Fukushima	0.9759
Gunma	0.9784
Shiga	0.9785
Wakayama	0.9789
Ehime	0.9798
Miyazaki	0.9801
Saga	0.9809
Yamagata	0.9810
Ibaraki	0.9826
Niigata	0.9828
Tottori	0.9836
Nagasaki	0.9844
Nagano	0.9846
Akita	0.9882
Kochi	0.9892
Iwate	0.9904
Kagoshima	0.9919
Kagawa	0.9925
Yamaguchi	0.9926
Yamanashi	0.9929
Toyama	0.9929
Tokushima	0.9930
Aomori	0.9933
Fukui	0.9940
Shimane	0.9996

## Data Availability

Not applicable.

## Appendix A

The 0–1 test procedure using the notation outlined by Gottwald et al. [21] is as follows:

***Step 1***. Define a time-series ***x(t)*** for ***t = 1,…,N***, where ***x(t)*** is the time-series value at time ***t***. Additionally, define a set C ∈ℜ of non-negative random values. Gottwald et al. [21] suggested random values for C in the interval (0, ***π***); therefore, in the present study, values for C are in the interval (π/5, 4π/5) to avoid resonances distorting the statistics. While c = 0 must be avoided, and exclusion of c =π is not necessary, the authors found the above procedure helpful.

***Step 2***. For each ***c***: ***c***∈ C, and ***n*** = 1, 2, …, ***N***, compute the translation variables ***p_c_ (n)*** and ***q_c_ (n)*** as follows:

(A1) $$p_{c}(n)=\sum_{t=1}^{n}x\left(t\right)cos\;tc,\;\mathrm{and}\;q_{c}(n)=\sum_{t=1}^{n}x\left(t\right)sin\;tc,\;\mathrm{for}\;n=1,2,\ldots ,\;N$$

After step 2, this procedure generates N sets ***p_c_ (n)*** and ***q_c_ (n)*** for each ***c***: ***c***∈ C considered.

***Step 3***. Compute the mean square displacement for the translation variables (i.e., Fourier coefficients) ***p_c_ (n)*** and ***q_c_ (n)*** as follows:

(A2) $$M_{c}(n)=\underset{N\;\to \infty }{\lim}\frac{1}{N}\sum_{t=1}^{N}[p_{c}\left(t+n\right)-p_{c}\left(t\right){]}^{2}+[q_{c}\left(t+n\right)-q_{c}\left(t\right){]}^{2}$$

According to Gottwald et al. [20,21], for the time-series with regular dynamics, the mean square displacement is a bounded function in time, whereas it scales linearly with time (***n***) if the time-series is chaotic. In such a case, the plot of ***p_c_ (n)*** vs. ***q_c_ (n)*** looks like an irregular not bounded function in time. Gottwald et al. [21] suggested that this definition of the mean square displacement requires that be much less than ***N*** or n << N. Gottwald et al. [21] also suggested a selection of ***n*** such that ***n<n_cut_***, where ***n_cut_*** = ***N***/10, and reported that this scheme of selecting n produced good results (plots) showing regular dynamics and chaotic dynamics clearly in a ***p_c_ (n)*** vs. ***q_c_ (n)*** graph.

***Step 4***. The ***M_c_(n)*** vs. ***n*** plot shows increasing linearity with ***n***; however, there is an oscillation confounded with ***M_c_(n)***. To smooth this oscillation, Gottwald et al. [21] suggested using the modified mean square displacement for the translation variables, which can be defined as follows:

***D_c_*** (***n***) = ***M_c_*** (***n***) – ***V***_osc_ (***c, n***) (A3)

where ***V***_osc_ (***c***, ***n***) = (Ex)^2^ $\frac{1-\cos nc}{1-\cos c}$ and Et = $\underset{N\;\to \;\infty }{\lim}\frac{1}{N}\;\overset{N}{\underset{t=1}{\sum }}x\left(t\right)$

***Step 5***. Finally, ***D_c_(n)*** s values from step 4 were used to estimate the asymptotic growth rate, ***K_c_***. Using the correlation method, the asymptotic growth rate ***K_c_*** can be calculated as follows:

(A4) $$K_{c}=\frac{cov\;\left(\xi ,\;\Delta \right)}{\sqrt{var\left(\xi \right)var\;\left(\Delta \right)}}\;\in \left[-1,1\right]$$

where **ξ** = (1, 2, …, n_cut_), ∆ = (D_c_(1), D_c_(2), …, D_c_(n_cut_)) and cov(**ξ**, ∆) is the covariance between vectors **ξ** and ∆. Finally, Gottwald et al. [29] also provided theoretical proof that their test yields a value of 0 for periodic and quasi-periodic dynamics and a value of 1 for nonlinear (chaotic) systems.
